# Looking at a baby's heart through the lens of the mother's blood

**DOI:** 10.15252/emmm.202318680

**Published:** 2023-10-16

**Authors:** Paolo Madeddu

**Affiliations:** ^1^ Bristol Medical School, Translational Health Sciences, and Bristol Heart Institute University of Bristol Bristol UK

**Keywords:** Biomarkers, Cardiovascular System, Development

## Abstract

Congenital heart disease (CHD) comprises several cardiovascular abnormalities existing from birth. Cardiac defects range from minor asymptomatic lesions to potentially life‐threatening situations. Early fetal echocardiography, the gold standard for the in‐utero diagnosis of CHD, is inaccurate at identifying defects in pulmonary veins and atrioventricular valves or lesions that occur later or progress during pregnancy. In this issue of *EMBO Molecular Medicine*, Yin *et al* report new proteomic data on maternal blood samples and a novel bioinformatic and artificial intelligence approach for the early diagnosis and screening of CHD.

## Graphical abstract

P. Madeddu discusses the potential of maternal blood proteomics for early diagnosis and screening of congenital heart disease as reported by JY. Zhao and colleagues, in this issue of EMBO Mol Med.

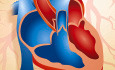

Critical advances in the diagnosis of CHD include genetic screening, fetal echocardiography, and visualization technologies, such as 3D printing, virtual reality, and augmented reality for surgical preplanning. Improved therapeutic results have been obtained thanks to the wider access to centers equipped to the care for vulnerable infants, the development of innovative pediatric anesthesiologic and surgical techniques for correction of complex CHDs, and effective postoperative follow‐up (Rao, 2023). Nevertheless, even forms that are classified as “simple,” like the ventricular septal defect (VSD), on the generic assumption of modest long‐term risks after surgical correction in childhood, reportedly carry a substantial burden of cardiovascular morbidity irrespective of defect correction (Eckerstrom *et al*, 2023).

Genetic and molecular biomarkers have been proposed to aid the early identification of CHD (Morton *et al*, 2022). These indirect methodologies provide preliminary evidence for the need of imaging‐based diagnosis of the disease. The sensitivity and congruency of CHD gene panels are modest, many of the proposed genes require further validation, and the proper translation and communication of candidate gene usefulness to end‐users is not well‐standardized (De Backer & Muino Mosquera, 2023).

Molecular biomarkers are also useful in predicting fetal CHD in utero. They identify transcriptional and post‐transcriptional alterations that are significantly more frequent in CHD than in the normal population. These tests can be applied to fetal or maternal tissues, cells, and biological liquid. An exemplar study from O'Brien *et al* identified the differential expression of a panel of noncoding microRNAs (miRs) and spliceosomal RNAs impacting on SOX4 and Notch developmental pathways in the myocardium of children with Tetralogy of Fallot compared with normally developing children (O'Brien *et al*, 2012). However, changes in protein levels are often not reflected by RNA levels. Therefore, major efforts have been focusing on the assessment of protein expression levels and interaction networks in the fetal heart. Proteomic‐based studies on fetal cells still face ethical and technical limitations, including the amount of material necessary for an in‐depth analysis of a protein interactome from cells and subcellular material and the complexity of miniaturizing the assay for clinical use (Dorr & Conlon, 2019).

To circumvent some of these issues, proteomic network analysis was applied to cardiovascular cells derived from embryonic stem cells (ESCs) and induced pluripotent stem cells (iPSCs), or direct reprogramming of differentiated cells (e.g., cardiac fibroblasts) into cardiomyocytes. A fundamental study from Dr. Srivastava's laboratory and Gladstone colleagues mapped out the entire network of interactions between the GATA4 and TBX5 proteins using human iPSC‐derived precursor heart cells. A 273‐protein network generated from DNA sequencing data from over 3,000 children with CHD, and their parents was investigated using a computational tool that ranks the candidates according to their likelihood of contributing to CHD (Gonzalez‐Teran *et al*, 2022). Another quantitative proteomics study investigated whether temporal changes in the proteome could affect murine embryonic heart development. Within a global temporal profile of the over 7,300 proteins, cardiac protein interaction networks revealed a functional role for the mevalonate pathway in regulating the cell cycle of embryonic cardiomyocytes (Edwards *et al*, 2023).

The detection of biomarkers in maternal blood and urine is attracting much attention for prenatal diagnosis. The main advantage over the analysis of fetal or placental tissues is the avoidance of risk‐associated invasive procedures, such as amniocentesis and chorionic villus sampling. Analytes emerging from the mass spectrometry screening could be measured using customized assays in primary hospital laboratories, thereby instructing the rapid referral of suspected cases to fetal echocardiography. Factors found to be differentially modulated in blood comprise maternal proteins, metabolites, and RNAs as well as molecules of fetal and placental origin. Placental dysfunction is frequently associated with and contributes to morbidity and mortality in CHD. Some of these markers have been already used to monitor the “placental proteomic clock” in at‐risk pregnancies and for the screening of diseases such as trisomy 21, which are at risk for CHD (Degnes *et al*, 2022; Guibourdenche *et al*, 2023).

In this issue of *EMBO Molecular Medicine*, Yin *et al* (2023) report the results of a case–control study on 206 pregnant women recruited from the International Peace Maternity and Child Health Hospital of the China Welfare Institute. The cohort comprised 103 controls and 104 cases, with the most frequent phenotypes being VSD (42/104) and atrial septal defect (ASD) (20/104). A proteomic analysis of plasma samples collected during the first trimester revealed significant differences between the controls and the CHDs. Among the proteins differentially modulated in CHDs, principal component analysis identified several members of amino acid and carbohydrate metabolism, extracellular matrix receptor pathway, actin skeleton regulation, and cardiac muscle contraction. Using machine learning, the authors generated a high sensitivity/specificity model, with an area under the receiver operating characteristic curve (AUC) of 0.964.

This new investigation follows recent studies focusing on the measurements of miRs and proteins in maternal blood. Two separate studies on 60 and 100 women, equally distributed between control and CHD pregnancies, identified distinct panels of miRs enriched in the CHD subgroups. Panel 1 included miR‐19b, miR‐22, miR‐29c, and miR‐375. (Zhu *et al*, 2013); Panel 2 comprised miR‐142‐5p, miR‐1275, miR‐4666a‐3p, and miR‐3664‐3p (Gu *et al*, 2019). The sensitivity of Panel 1 ranged from 56 to 78% and specificity from 67 to 89%, with further improvements when considering the combination of the four differentially expressed biomarkers. There was also a trend for each miR to associate with a specific defect. The second study included a follow‐up phase on 10 women at gestational ages between 38 and 40 weeks, measuring the candidate miRs 24 h after normal delivery. The authors found that the expression levels of all miRNAs dropped off significantly after delivery (Gu *et al*, 2019). It would have been valuable to confirm these miRs using a blinded protocol in samples collected before 20 weeks’ gestation.

In the proteomic arena, a study examining pooled serum from 370 women used isobaric tagging for relative and absolute proteomic quantification. Forty‐seven proteins displayed significant differential expression and targeted verification using multiple reactions monitoring mass spectrometry. Validation by ELISA eventually defined a panel composed of four cytoskeletal proteins with very high diagnostic ability in differentiating CHD‐pregnancies from normal ones, as indicated by an area under the receiver operating characteristic curve (AUC) of 0.938 (Chen *et al*, 2016).

Another case–control study compared biomarkers in the urine of women whose infants were diagnosed with CHD (70 case subjects) or were healthy (70 control subjects) using the modified gas chromatograph‐mass spectrometry method. The top biomarkers associated with CHD were 4‐hydroxybenzeneacetic acid, 5‐trimethylsilyloxy‐n‐valeric acid, propanedioic acid, hydracrylic acid, and uric acid, which was interpreted with the fact the short‐chain fatty acids and aromatic amino acid metabolism may be relevant with CHD (Xie *et al*, 2019).

There are several elements of originality in the study from Yin *et al* (2023). Apart from confirming plasma proteins already known for being associated with CHD, the authors identified other significantly changed proteins, such as deoxynucleoside triphosphate triphosphohydrolase 1 (SAMHD1) and secreted protein acidic and rich in cysteine (SPARC). A panel of 74 proteins differentially regulated were consistent between groups recruited at different clinical sites. Probably, the most tantalizing finding in Ya‐Nan Yin's paper is the matching of protein modules, and related metabolic and cellular landscapes, with three clusters of different anatomical severity. Since the majority of recruited cases comprised ASD and VSD, one may speculate that common proteomic features intercept a general defect in the early heart formation.

Whether the early protein modules diverge during fetal maturation remains to be ascertained through a longer follow‐up of the hierarchical clustering analysis. Adoption of a labeled machine‐learning strategy, tested already in this study but necessarily requiring validation in a much large cohort, could lead to a high accuracy classification of different CHD groups. Unsupervised learning models could be also advantageous for handling very large volumes of data in real time.

As with all innovative studies, this one carries limitations, questions, and controversies. The authors anticipate that CHD detection in underdeveloped regions is problematic because of low accessibility to specialistic hospitals. The introduction of a maternal blood test within a Point‐of‐Care Technology plan may aid the screening of CHD in poor regions. Improvement in patient outcomes would still depend on whether the local healthcare system has the infrastructure to translate the data into better clinical outcomes (Mitra & Sharma, 2021).

Prenatal diagnosis of complex CHD helps families to decide whether to continue with pregnancy and allows swift referral of CHD babies to specialistic centers for delivery and corrective surgery planning. Early diagnosis should increase the survival rate and decrease the rate of complications. A recent review article highlighted the controversial evidence for prenatal diagnosis ability in reducing neonatal mortality and morbidity. Moreover, a rigorous comparison of the cost/benefit of performing or not a prenatal diagnosis using indirect methods such a maternal blood proteomics is still missing (Bonnet, 2021). While the translation of maternal blood proteomics to the routine clinical practice might prove to be a long journey, this new proteomic study reveals the precious value of maternal blood in generating novel mechanistic insights of the baby's developing heart.

## Disclosure and competing interests statement

The authors declare that they have no conflict of interest.
